# Postural and Lumbopelvic Control: Crucial Factors in the Functionality of Patients with Low Back Pain—A Descriptive Cross-Sectional Study

**DOI:** 10.3390/jcm13133836

**Published:** 2024-06-29

**Authors:** Katherine Stöwhas, Guillermo Droppelmann, Carlos Jorquera, Felipe Feijoo

**Affiliations:** 1 Department of Rehabilitation, Clínica MEDS, Santiago 7691236, Chile; katherine.stowhas@meds.cl; 2 Facultad de Medicina, Escuela de Kinesiología, Universidad Finis Terrae, Santiago 7501014, Chile; 3 Harvard T.H. Chan School of Public Health, Boston, MA 02115, USA; 4 Facultad de Ciencias, Escuela de Nutrición y Dietética, Universidad Mayor, Santiago 8580745, Chile; carlos.jorquera@mayor.cl; 5 School of Industrial Engineering, Pontificia Universidad Católica de Valparaíso, Valparaíso 2362807, Chile; felipe.feijoo@pucv.cl

**Keywords:** functionality, low back pain, lumbopelvic control, pain, postural control

## Abstract

**Background:** Low back pain (LBP) is one of the most prevalent musculoskeletal disorders in adults worldwide. Alterations in postural and lumbopelvic control and functionality appear to be determining factors in its resolution. **Methods:** A cross-sectional study was performed. Patients with LBP were enrolled. Lumbar pain; postural control (PC), total area of the center of pressure (TACOP), and the velocity of the center of pressure (VCOP); lumbopelvic control (LPC); and functionality were evaluated. Statistical tests were implemented to determine differences between sex and age and correlation models among the variables. **Results:** Thirty adult patients with LBP were analyzed. A strong relationship was found between pain and functionality [r = 0.64; *p* < 0.001]. A moderate relationship was found between pain and TACOP [r = 0.395; *p* = 0.031]. A moderate relationship was observed between TACOP and functionality [0.413; *p* = 0.023] and between LPC and TACOP [r = 0.416; *p* = 0.001]. **Conclusions:** This study demonstrates the significant impact of LBP on postural control, lumbopelvic control, and functionality. These results highlight the importance of addressing postural and lumbopelvic control in LBP treatment. No significant differences based on gender and age were found, but all clinical variables differed significantly between the LBP and control groups, underscoring the unique impairments associated with LBP.

## 1. Introduction

Musculoskeletal pathologies represent one of the main problems and challenges for public health worldwide [1,2]. More than 1.710 million people suffer from some musculoskeletal disorder. In 2019, low back pain syndrome (LBP) reached second place with an incidence of 568 million people per year [3]. A year later, a global burden of disease report stated that the condition affected approximately 620 million individuals, projecting the figures to surpass 840 million people by the year 2050 [4].

In Latin America, there is limited information and methodological heterogeneity across studies. A high prevalence has been indirectly estimated, consistent with international reports [5]. This is especially true among young adults and vulnerable populations such as women, indigenous people, those with lower educational levels, and individuals with comorbidities [6].

Particularly in Chile, the prevalence of musculoskeletal pain is 41.1%, with half of these cases (21.1%) attributed to LBP [7]. The annual cost of musculoskeletal chronic pain was estimated at USD 1387.2 million, equivalent to 0.417% of the national gross domestic product (GDP). Additionally, lumbar low back pain (LBP) generated the highest costs and disability rates [8]. This makes it the leading disease burden in our country. Although mortality rates for this condition are low compared to other diseases, its high morbidity burden and associated costs position it as a significant public health issue [9,10]. Around 20% of individuals who have experienced an episode of acute low back pain will develop chronic low back pain, leading to increased disability and directly impacting people’s quality of life [11,12].

The origin of the pain is linked to a variety of factors, among which biological, psychological, social, behavioral, and learning-related factors stand out, all of which influence its chronicity [13,14]. The correlation between low back pain and functionality is a widely researched topic, leading to the conclusion that the relationship between pain and dysfunction is poor. The understanding of low back pain is limited and found to be mediated by a series of biopsychosocial factors, with little connection to any physiological or pathological changes [15,16]. Researchers have extensively studied the relationship between postural alterations and pain [17,18]. The alteration of postural control (PC) is one of the key biological factors associated with low back pain. This is explained by the central nervous system’s inability to accurately process sensory input [19]. This affects the ability to generate appropriate motor output, perpetuating both pain and instability [20,21]. Therefore, we hypothesize that measuring factors such as displacement area or total area of the center of pressure (TACOP) and velocity of the center of pressure (VCOP) could help us better understand the relationships with other clinical variables affecting neuromuscular control and dynamic stability of the spine in subjects with LBP.

On the other hand, lumbopelvic control (LPC) has been studied as a key element in segmental stabilization of the spine [22,23]. The presence of lumbopelvic instability is implicated in the onset and chronicity of pain, as well as the dysfunction of patients with low back pain [24].

While the relationship between the presence of alterations in PC, LPC, and pain has been described, it is not clear to what extent these variables can be measured in a clinical setting using validated and easily implemented instruments to determine the degree of dysfunction in patients with LBP. The objective of the present study is to explore differences by sex and age and between healthy subjects and those with LBP, as well as the relationship between pain, PC, TACOP, VCOP, LPC, and functionality in patients with LBP. The results of this study could address critical gaps in the practical application of clinical measurements for LBP, enhance the understanding of demographic influences on LBP, and contribute to the scientific literature on neuromuscular control and functionality. The outcomes have the potential to improve diagnostic accuracy, personalize treatment approaches, and ultimately lead to better patient outcomes in managing low back pain.

## 2. Materials and Methods

### 2.1. Ethics Statement

All included participants provided written consent prior to clinical evaluation and data collection. This study was conducted with the approval of the Scientific Ethics Committee, Finis Terrae University, ‘Comité Ético Científico, Universidad Finis Terrae’, Santiago de Chile, Chile. The approval date was in August 2022, and it began after approval. The approval ID protocol was 22-044.

### 2.2. Study Design

This descriptive cross-sectional study was conducted at a single healthcare institution. The STROBE (Strengthening the Reporting of Observational Studies in Epidemiology) checklist was followed (https://www.strobe-statement.org/ (accessed on 26 June 2024).

### 2.3. Participants

Between August 2022 and August 2023, participants were recruited. Orthopedic spine surgeons certified and approved by the Chilean Orthopedics Society evaluated individuals of both sexes, over 18 years of age, who initiated physical therapy treatment due to a diagnosis of low back pain at the MEDS La Dehesa Clinic in Santiago, Chile. Orthopedic spine surgeons certified and approved by the Chilean Orthopedics Society diagnosed LBP. A group of healthy volunteers without LBP was similarly recruited as the control group.

### 2.4. Sample Size 

A convenience sampling method was employed. The estimation of the number of subjects to be evaluated was conducted using predefined criteria based on recent publications [25]. Recruitment was carried out sequentially, considering selection criteria and obtaining informed consent.

### 2.5. Selection Criteria

The inclusion criteria were signed informed consent and both sexes between 18 and 60 years old. For the LBP group, the selection criteria included patients diagnosed with lumbar degenerative facet and intervertebral disc pathology between segments L4–L5 and L5–S1, confirmed by MRI, and experiencing low back pain for more than 1 month, as well as pain intensity ≥2 on the visual analog scale (VAS), indication for rehabilitation, and the Oswestry Disability Index (ODI) functionality index at any level of classification. The exclusion criteria were patients with surgical referrals; a history of surgery or infiltration in the spine and/or lower limbs; balance or vestibular, neurological, sensory, and lower limb strength alterations; the presence of radiculopathy; pain in the lower limbs; and incomplete information.

For the control group, the selection criteria included no self-reported neurological or musculoskeletal issues, pain, or disabilities for at least 6 months before evaluation. Individuals with a history of low back pain, neck or lower extremity injuries within the past 6 months, known balance problems, or those taking pain-suppressing or sensory-altering medications were excluded. The control group’s physical examination also had to show no back or extremity complaints or significant biomechanical impairments that could affect the measurements.

### 2.6. Personal Variables

The variables of sex and age of the participants were identified. Additionally, age was divided into the categories [20–40 years] and [41–60 years]. Anthropometric variables such as weight and age were recorded, and a calculation of the body mass index (BMI) was performed. The level of physical activity was assessed using the International Physical Activity Questionnaire Short Form (IPAQ-SF). This international questionnaire consists of 7 questions about the frequency, duration, and intensity of activity performed in the last seven days, as well as walking and time spent sitting on a workday. Values in METs were obtained, allowing the classification of the level of activity into three categories: low, moderate, and high [26]. The assessments of LPC and PC were conducted by a physical therapist specializing in spinal dysfunctions with over 20 years of experience.

### 2.7. Assessment of Low Back Pain Intensity

All patients were assessed during the first treatment session by a physiotherapist specializing in musculoskeletal disorders with over 20 years of experience in spinal dysfunctions. To avoid assessment bias, the professional was not included in the research team. The evaluation of LBP was conducted using the visual analog scale (VAS) [27,28], with pain rated on a 100 mm line; 0 mm corresponded to no pain, and 100 mm indicated the maximum unbearable pain. Each patient marked the intensity of the pain on the horizontal line, and it was measured from the beginning of the line to the point marked by the patient using a millimeter ruler to quantify the intensity.

### 2.8. Assessment of Lumbopelvic Control (LPC)

The Active Straight Leg Raise (ASLR) test [29,30] and a pressure biofeedback unit (Stabilizer^®^, Chattanooga Group, Inc., Hixson, TN, USA) were used in the lumbar region. This test has a good intraclass correlation coefficient (ICC) of 0.74 for intra-examiner reproducibility and 0.76 for inter-examiner reproducibility [31]. The Stabilizer is a reliable, non-invasive device that combines an inflation manometer connected to a pressure cell. It records changes in a pressure-filled air cell during various movements of the lower limbs, allowing the determination of neutral pelvic alignment during active extension of the extended leg [32,33].

**Figure 1:**
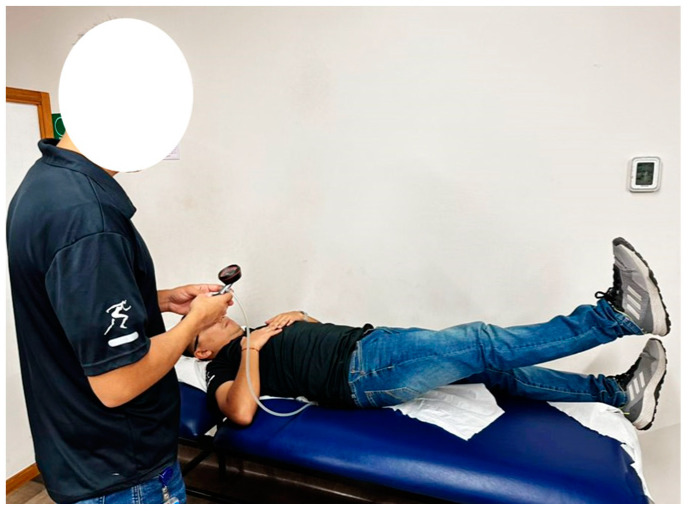
Lumbopelvic control assessment.

The ASLR test was performed in a supine position according to the authors’ recommendations [32]. The inflatable pad was placed horizontally under the participants’ lumbar spine, with the lower edge at the level of the posterior superior iliac spines and inflated to 40 mmHg. One extended lower limb was raised 20 cm and held for 10 s. ASLR tests were conducted for elevation of both lower limbs. Each test was repeated three times, and the mean value was recorded. See Figure 1.

### 2.9. Assessment of Postural Control (PC)

**Figure 2:**
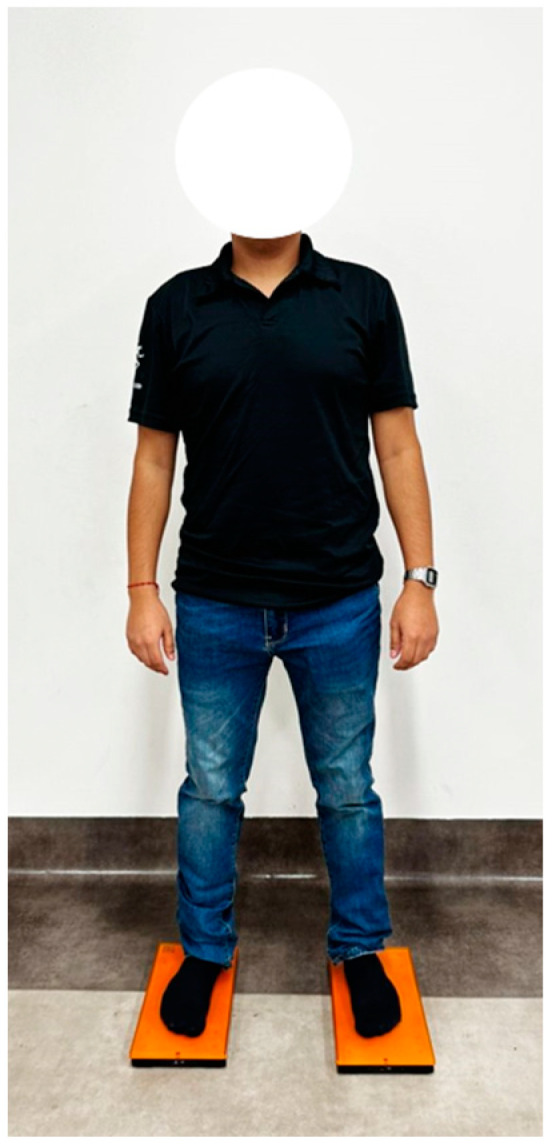
Posturography in bipedal balance.

The PC was evaluated using posturography in bipedal balance with closed eyes [34]. The patients stood on the platform with one foot on each plate, which were separated by the width of the hips. Once on the platforms, they closed their eyes with their heads upright and arms hanging at their sides for 30 s. See Figure 2.

**Figure 3:**
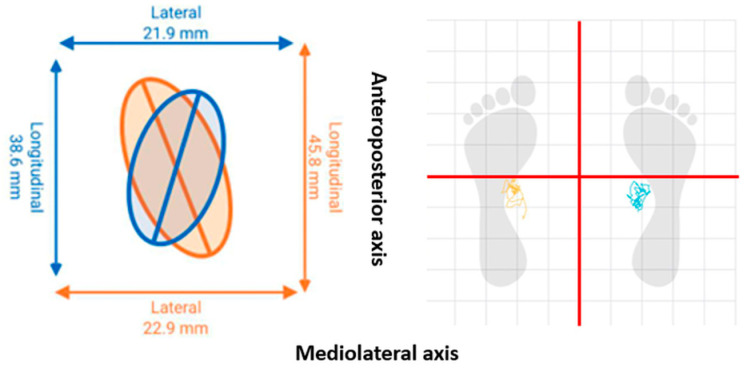
Total displacement of the center of pressure.

Total displacement values of the center of pressure (COP) were determined using the total area of the center of pressure (TACOP) (mm) and the velocity of the center of pressure (VCOP) (mm/s). COP parameters showed high to very high test–retest reliability, with an ICC range of 0.74–0.91 [35]. The PLATES v3 force platform (K-invent, Montpellier, France) was used as a tool that quantifies the COP on a static support base. The information was collected via Bluetooth connection using the KINVENT PHYSIO application, Montpellier, France. The platform provided the information detailed in Figure 3.

### 2.10. Functionality Levels: Oswestry Disability Index (ODI)

The Oswestry Disability Index is a valid instrument for evaluating the management of spinal disorders [36]. It represents one of the most common measures for assessing low back pain. This self-administered questionnaire requires five minutes to complete and one minute to obtain a score. Scores are associated with the degree of disability using a Likert scale (minimal, moderate, severe, crippled, bed-bound) [37]. Currently, it remains one of the most widely used due to its high consistency in psychometric analyses [38].

### 2.11. Statistical Analysis

Descriptive and inferential statistics were applied. Normality assumptions were assessed using the Shapiro–Wilk test. The homogeneity of variance was evaluated using Levene’s test. To check for differences in the variables among sex, age group, and between groups, Mann–Whitney T and U tests were used. 

To assess the reliability of the LPC and PC measures in this study, we computed the two-way random effect intraclass correlation coefficient (ICC(2, k)) using absolute agreement. Additionally, we calculated the 95% confidence intervals (CI) and the standard error of measurement (SEM) [39]. For this study, we interpreted the ICC values according to the following criteria: values less than 0.5, between 0.5 and 0.75, between 0.75 and 0.9, and greater than 0.90 are indicative of poor, moderate, good, and excellent reliability, respectively [40].

The relationship between variables such as BMI, physical activity, pain level, LPC, TACOP, VCOP, and levels of functionality in the LBP group was explored. The mean (SD) was obtained, and a one-way ANOVA was applied for multiple comparisons, with results adjusted using the Bonferroni test. Finally, Pearson and Spearman correlation analyses were conducted. Correlation was considered weak if ∣r∣ ≤ 0.3, moderate if 0.3 < ∣r∣ ≤ 0.7, and strong if ∣r∣ > 0.7. The level of statistical significance was set at *p* ≤ 0.05. All data were analyzed using IBM SPSS Statistics 28 (IBM Corp., Armonk, NY, USA) and R statistical software (R version 4.3.1).

## 3. Results

### 3.1. Participants

A total of 60 subjects completed this study. Thirty patients with LBP and thirty healthy control subjects successfully completed the PC and LPC tests without difficulty. Of the total subjects, 18 (60%) were male in both groups. The mean age was 41.2 (±12.9) years for the LBP group and 42.26 (±2.88) years for the control group. The mean BMI was 24.8 (±3.1) kg/m^2^; for the LBP group and 24.26 (±0.56) kg/m^2^ for the control group, with no significant differences between them (*p* = 0.407). Additionally, a significant difference in BMI was observed between men and women in the control group with a *p*-value of 0.003.

**Figure 4:**
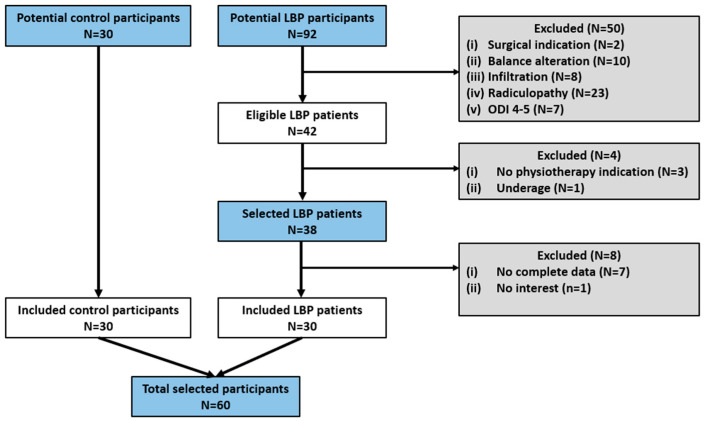
Participant flow.

Regarding personal variables related to work and level of physical activity, all participants are professionals who are not subjected to daily tasks involving high-demand activities that could affect the lumbar spine. The average time spent daily in a seated position was 6 h, and the mean weekly physical activity recorded in METs was 1540 for men and 961 for women classified as moderate in the LBP group, and 4852 for women and 3697 for men classified as high in the control group. No significant differences were found when comparing by sex in the LBP (*p* = 0.24) and the control group (*p* = 0.31), but significant differences were found when comparing each sex by groups (*p* < 0.05). Interestingly, unlike the LBP group, women in the control group were found to be more active than the men, and consequently, they presented a lower BMI. The flow of participants is represented in Figure 4.

For the assessment of reliability of the LPC and PC measures, we analyzed the averages of three assessments for both tests, which allowed us to achieve excellent and good reliability in the LPC and COP parameters, respectively. Further information is available in Table 1.

When analyzing the differences in clinical variables, no significant differences where *p* > 0.05 were observed between the variables BMI, physical activity level, pain, LPC, TACOP, VCOP, and functionality based on sex categorization in both the LBP and control group, except for BMI, where significant differences between sexes were observed in the control group with a *p*-value of 0.003. Further information is available in Table 2.

On the other hand, no significant differences where *p* > 0.05 were observed between almost all the same clinical variables based on age categorization in both groups. There is only a significant difference in the functionality variable in the control group with a *p*-value of 0.038, where individuals in the older age group (41–60) showed greater dysfunction than the younger. See Table 3.

**Figure 5:**
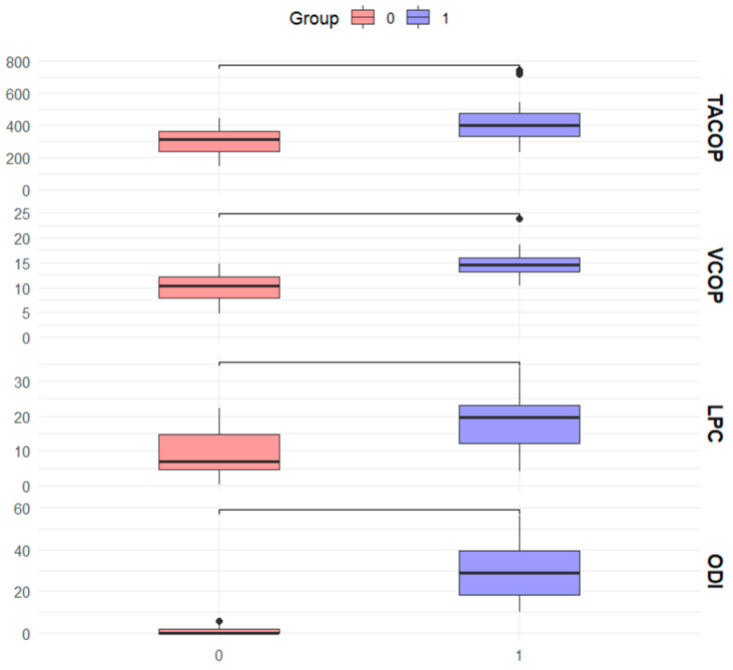
Differences between control (0) and LBP (1) group.

The results for the variables show significant differences between the two groups. For TACOP (mm), the first group had a mean value of 422.3 ± 24.3 mm, while the second group had a mean value of 307.1 ± 18.4 mm, with a *p*-value of 0.001, indicating statistical significance. In VCOP (mm/s), the first group presented a mean value of 18.6 ± 8.4 mm/s, compared to 9.01 ± 6.6 mm/s in the second group, with a *p*-value of <0.001, also statistically significant. For LPC (mmHg), the first group had a mean value of 14.8 ± 2.7 mmHg, while the second group had a mean value of 10.2 ± 2.7 mmHg, with a *p*-value of < 0.001, showing a significant difference. Lastly, functionality (ODI) showed a mean value of 31.1 ± 0.5 in the first group and 1.2 ± 0.4 in the second group, with a *p*-value of <0.001, indicating a significant difference between the groups. See Figure 5.

In this sense, our results indicate significant differences in the variables of pain (VAS), postural control (TACOP and VCOP), lumbopelvic pressure (LPC), and functionality (ODI) between patients with low back pain and the control group. All subgroups showed a very large effect size (Cohen’s d > 4.0) with a statistical power of 1.0 in the VAS, indicating very significant differences and high probabilities of detecting these differences between patients with LBP and the control group. The effect size varied among subgroups in TACOP. Males with LBP (Cohen’s d = 1.35) and patients aged 20–40 years with LBP (Cohen’s d = 1.27) showed large effect sizes with high statistical power (0.99). Females with LBP (Cohen’s d = 1.07) also showed a large effect size with a statistical power of 0.98. However, the 41–60 years age group showed a moderate effect size (Cohen’s d = 0.78) with a lower statistical power (0.84). Also, all subgroups showed very large effect sizes (Cohen’s d > 1.5) with a statistical power of 1.0 in VCOP, indicating very significant differences and high probabilities of detecting these differences between patients with LBP and the control group. The effect sizes for the subgroups were large in LCP, with males with LBP (Cohen’s d = 1.22), females with LBP (Cohen’s d = 1.33), and the 41–60 years age group (Cohen’s d = 1.51) showing statistical powers of 0.99. The 20–40 years age group showed a moderate effect size (Cohen’s d = 1.08) with a statistical power of 0.98. Finally, all subgroups in ODI showed very large effect sizes (Cohen’s d > 2.8) with a statistical power of 1.0, indicating very significant differences and high probabilities of detecting these differences in functionality between patients with LBP and the control group. See Table 4.

### 3.2. Clinical Variables Based on the Degree of Disability in the LBP Group

There are no differences in the BMI and physical activity level variables associated with the level of disability. Although there is a decrease in physical activity level as disability increases, the differences between the three categories are not statistically significant (*p* = 0.66) Statistically significant differences are observed in the rest of the clinical variables. Out of the total patients, 7 belonged to the mild disability category, 14 to moderate, and 9 to severe. Subjects with mild disabilities had a mean of 4.1 points in the pain variable, which increased as the degree of disability increased, reaching a mean of 6.57 in severe cases. Subjects with mild and severe disabilities showed significant differences in pain scores, with a *p*-value of 0.001.

Pressure changes increased as disability levels rose, with severe cases reaching a mean of 21.85 mmHg. However, these differences were not significant among the disability groups *p* > 0.05.

The PC variables followed the same pattern as the previous ones, with increases in TACOP and VCDP as the subjects’ disability increased. Patients with severe disability showed a significant increase of almost 200 mm in the COP displacement area compared to those with mild disability, with a *p*-value of 0.015. See Table 5 for more details.

### 3.3. Correlation Models

The relationship between pain and LPC was [r = 0.286; *p* = 0.125]. This correlation is weak and not significant, suggesting that although there are high pain values above seven points, the changes in LPC are low and around 20 mmHg, and there is high variability in LPC values for the same degree of pain. On the other hand, the relationship between pain and TACOP was [r = 0.395; *p* = 0.031], showing a moderate and significant correlation. However, the relationship between pain and VDCP was [r = 0.234; *p* = 0.214], resulting in a weak and non-significant correlation.

It is important to note that high values on the VAS scale above seven points are associated with greater displacement of the COP, exceeding 500 mm, while the same trend is not observed with velocity. Additionally, a strong relationship was found between pain and functionality [r = 0.64; *p* < *0*.001]. It was identified that patients with severe pain and scores above 7 points on the VAS scale had functionality values above 40 points on the ODI scale, reflecting severe disability associated with high pain.

A moderate and significant correlation was also observed between LPC and TACOP [r = 0.416; *p* = 0.001], but it was weak and not significant with VCOP [r = 0.308; *p* = 0.098]. Furthermore, it is highlighted that LPC values above 20 mmHg are related to higher degrees of COP displacement, exceeding 400 mm. In contrast, values below 20 mmHg mostly do not exceed 350 mm of COP displacement. Regarding the level of relationship between LPC and functionality [r = 0.364; *p* = 0.048], although it was significant, it was the weakest relationship. ASLR values above 22 mmHg were linked to increased dysfunction.

**Figure 6:**
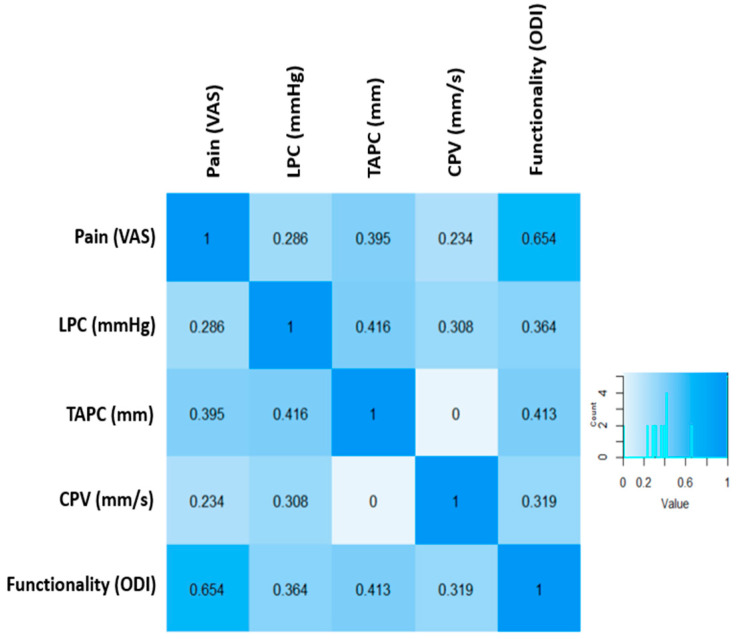
Correlation between clinical variables in LBP.

The TACOP with functionality [0.413; *p* = 0.023] showed a moderate and significant correlation. In this case, elevated COP displacement values above 400 mm were linked to higher degrees of dysfunctionality, with scores exceeding 41 points on the ODI scale, representing severe disability. On the other hand, the same behavior was not observed with VCOP and functionality [r = 0.319, *p* = 0.86], where patients with elevated COP displacement values above 15 mm had dysfunctionality levels below 20 points, indicating mild disability. The relationship between all analyzed variables is presented in Figure 6.

## 4. Discussion

In this study, we explored differences in an LBP group and a control group by sex and age, as well as the relationship between pain, PC, LPC, and the functionality of patients with LBP. The purpose was to estimate which variables are clinically relevant for the medical and rehabilitation teams. No differences were observed between clinical variables based on gender and age in both groups, except for the functionality variable segmented by age in the control group, where the older group showed a higher degree of dysfunction than the younger. Also, significant differences between the variables of physical activity level, pain, LPC, TACOP, VCOP, and functionality among subjects with LBP and those in the control group were observed. Additionally, we found a relationship between pain, PC, LPC, and the level of functionality in patients with LBP. The relationship observed between TACOP, LPC, pain, and functionality is particularly interesting, because elevated levels in pain and dysfunction parameters, as well as poor lumbopelvic control, are directly related to greater displacements of the center of pressure, reflecting alterations on standing balance control.

The results obtained in this article regarding sex differences in PC variables in patients with LBP are consistent with findings reported by other authors [41], establishing that there is no difference in static and dynamic postural control between men and women. However, they found that kinesiophobia and pain intensity during activity are more closely related to deteriorated dynamic balance in women with nonspecific chronic low back pain. Studies have reported no differences in PC among healthy populations based on sex [42], while others have established that women exhibit lesser motor control compared to men [43]. Researchers have not yet studied these differences in patients with LBP. The same applies to pain perception and functionality, where there is evidence of differences between both sexes [36,44,45,46] that were not reflected in the present study.

It is worth noting that possible variations in PC and LPC parameters between men and women should be considered when developing rehabilitation protocols. Careful intervention selection and dosage are critical for effective treatment strategies. A recent review [47] highlighted the importance of considering sex differences in the assessment, diagnosis, or treatment of low back pain.

However, this study did not observe differences in physical activity levels associated with gender; significant differences were found between subjects with and without low back pain. Patients without pain had a higher level of vigorous physical activity according to the IPAQ questionnaire, compared to the group with lumbopelvic pain, where activity levels were lower. It is important to consider that the IPAQ test considers only the activity performed during the last 7 days, which may have been reduced due to the presence of pain in the LBP group. This is why it is crucial to consider the level of physical activity when planning rehabilitation protocols by asking about physical activity levels before the onset of pain. This helps focus therapeutic strategies toward a sports return that meets the patient’s expectations.

Moreover, various studies in healthy subjects have demonstrated age-related differences in PC [48,49]. Researchers have also described this condition in patients with LBP. Studies have identified that older patients show greater alterations in PC [50]. After segmenting the analysis by age, we found no differences in PC. Similarly, there were no differences in pain, LPC, or functionality associated with age in patients of both groups, except for the functionality variable, which was lower in the older subjects of the control group. This is consistent with findings from another study [51] where PC parameters were compared between two groups of patients (20–35; 36–50 years old) with low back pain, showing no significant differences between them.

Report [52] describes a “paradoxical” pattern of age effects in patients with LBP, challenging the expectation of higher dysfunctionality in older individuals with low back pain. These reports establish that, although disability in patients with LBP increases with age, quality-of-life indicators are equal to or even higher than in younger patients. This is because older patients have lower functionality. Therefore, individuals experiencing LBP do not have their activities diminished, unlike younger individuals, for whom low back pain might affect participation more severely. In the case of healthy subjects, the intensity of pain was 0 for the entire group. When filling out the functionality questionnaire, older individuals may have experienced minimal alterations in some functions due to other factors and not necessarily because of age. Also, in this study, a sample of adults up to 60 years old was selected, considering possible alterations this population might have in PC and functionality. Therefore, the absence of significant differences in the studied parameters associated with age suggests the need to include a future population with a wider age range to better assess differences.

After analyzing the differences in the clinical variables of pain, PC, LPC, and functionality between the low back pain and control groups, it was observed that all variables had significant differences. Subjects with low back pain exhibited poorer postural and lumbopelvic control, as well as greater dysfunction. The neurophysiological pathways of pain can explain this, as pain preferentially localized in the lower back or leg can induce alterations in postural control, likely due to proprioceptive and cortical excitability modifications [53]. Our results are consistent with those obtained in the systematic review from Berenshteyn et al., which suggest that balance is impaired in individuals with chronic low back pain when compared to healthy individuals [17].

When comparing the variables stratified by levels of disability, an increase in values was observed as the degree of disability increased, demonstrating how alterations in pain, PC, and LPC are associated with greater disability in patients with LBP. Although all variables showed an upward trend with increasing disability, significant differences were only observed for pain and the total area of COP displacement between the groups with mild and severe disability. This reaffirms current theories associating increased pain with changes in CP [54].

Functionality, pain, LPC, and COP displacement showed a significant correlation in the analysis of the observed clinical variables. Researchers have extensively studied the alteration of CP in patients with LBP in recent years. A systematic review [55] concluded that patients with low back pain exhibit greater postural instability than healthy controls, manifested by larger COP excursions and a higher average COP displacement velocity. Additionally, they establish that, while decreased postural stability in subjects with LBP appears to be associated with the presence of pain, no correlation was identified between pain intensity and the magnitude of COP excursions. The present study did not compare the studied population to a group of healthy subjects to determine differences associated with pain. Patients with LBP have been extensively studied in recent years regarding the positive correlation found between pain and increased COP displacement, but not with velocity.

These results align with findings in the literature [56], indicating a close relationship between COP parameters and pain levels. Similarly, LPC also exhibits a correlation with pain. Both alterations are reflected in the results of the present study, reaffirmed by the existence of a significant difference in the values of LPC and PC between groups, where patients with pain show less control compared to healthy individuals without pain. This reinforces the association between pain and alterations in motor control. Injury or nociception can directly interfere with motor control by altering the excitability of motor pathways at various levels of the nervous system. This may explain some of the changes and variability in muscle activation observed in patients with LBP [57], which appears to cause reduced precision in the control of trunk movement [58]. Current theories suggest that pain induces changes across various levels of the motor system, altering mechanical behavior to protect affected tissues. While these changes may offer short-term benefits, they can be detrimental in the long run by disrupting the proper synergy of stabilizing musculature crucial for motor control [59]. Ultimately, these motor control alterations create an overload on the spinal structures, perpetuating pain and subsequently affecting the functionality of this population.

Lumbo-pelvic instability, often associated with pain, significantly impacts the functionality of individuals with LBP, leading to aberrant movement patterns that can exacerbate the condition over time. Although no strong association was found between pain and LPC, a notable correlation was observed with functionality, particularly evident in the strong correlation between LPC and COP displacement area. This underscores the direct influence of LPC alterations on standing balance control, emphasizing the need for treatment strategies aimed at restoring appropriate muscle activation patterns to achieve segmental spine stabilization [60,61,62].

The relatively low pain intensity reported by patients in this study may account for the differences observed compared to other studies, where strong associations were found between pain and LPC, as well as COP displacement velocity. Most patients reported moderate to low pain intensity, with a mean score of five points. A study [51] examining the correlation between pain intensity and CP found that subjects with moderate to low pain intensity did not exhibit significant balance alterations. This supports the theory that neurophysiological alterations due to pain may only impact COP measures at moderate to high pain intensities [63].

Finally, we highlight an article that evaluated 25 potentially meaningful functional outcomes, considering objective functional measurements such as trunk range of motion, dynamic and static balance, strength, and muscle fatigue resistance, as well as body characteristics [64]. Interestingly, the authors presented a significant BMI among the participants, but in our study, this variable was not decisive, presumably because our patients performed some level of physical activity, which could have been an influential variable in the results. However, it is striking that the researchers evaluated the COP, obtaining non-significant results, which is very contrary to the results presented in this study. This is because the researchers evaluated this condition with eyes open for 10 s, while we performed our intervention with eyes closed for 30 s and three repetitions to avoid any potential evaluation bias. We suggest that future studies consider this variable in different contexts to gain a deeper understanding of its behavior.

Several limitations were encountered during the development of this article. The small sample size hindered differentiation in pain levels or grouping by intensity ranges. Additionally, this study did not record pain temporality as a limiting factor in the performance of the evaluated tests, which would have been valuable information. This could have helped determine if chronic pain affects clinical parameters such as stability and postural control. Further research across diverse population scenarios is needed to assess pain, PC, LPC, and functionality in patients with LBP, allowing for the development of comprehensive treatment strategies. The prognosis of nonspecific low back pain is greatly influenced by factors unrelated to the spine, such as psycho-emotional factors [65]. The evaluation conducted in this study did not consider the psycho-emotional aspects of patients with LBP, which are crucial given the multifactorial nature of the dysfunction. This is why it is important to consider these variables in future studies, as they are determinants of the onset and chronicity of the clinical condition. Therefore, when treating patients with low back pain, clinicians should consider all aspects of the condition, including biomechanical, psychological, and psychosocial factors, through a biopsychosocial approach. It is essential to recognize that each patient is unique, and the approach to musculoskeletal dysfunction should be tailored to meet the needs and expectations of each individual.

## 5. Conclusions

The results of this study highlight the importance of considering the relationship between pain intensity, postural control, lumbopelvic control, and functionality in patients with low back pain. These findings emphasize the significant impact of LBP on postural and lumbopelvic control, as well as overall functionality, underscoring the need to address these aspects in LBP treatment. While no significant differences based on gender and age were found, all clinical variables differed significantly between the LBP and control groups, highlighting the unique impairments associated with LBP. These results suggest the importance of developing comprehensive therapeutic interventions that target these aspects. The study also indicates the need to thoroughly evaluate the total area of the center of pressure, as it appears to be a key factor strongly linked with most variables. Further research in diverse populations with LBP is recommended, using these and other assessment tools, to continue validating and expanding knowledge in the musculoskeletal clinical area.

## Figures and Tables

**Table 1:** Reliability of COP and LPC measures.

Variables	(ICC(2, k))	95%CI
LPC (mmHg)	0.974	0.95–0.98
TACOP (mm)	0.758	0.39–0.91
VCOP (mm/s)	0.76	0.40–0.91

TACOP, total area of the center of pressure; VCOP, velocity of the center of pressure; LPC, lumbo-pelvic control; (ICC(2, k)), two-way random-effect intraclass correlation coefficient; 95%CI, 95% confidence intervals.

**Table 2:** Clinical variables by sex across groups.

Variables	LBP Group			Control Group		
	Male (*n* = 18) Mean ± SD	Female (*n* = 12) Mean ± SD	*p*-Value (<0.05 *)	Male (*n* = 18) Mean ± SD	Female (*n* = 12) Mean ± SD	*p*-Value (<0.05 *)
BMI	25.6 ± 2	23.5 ± 3.9	0.065	26.2 ± 1.9	22.7 ± 2.5	0.003 *
IPAQ	1539.7 ± 332	960 ± 313	0.240	3697 ± 731	4852.2 ± 847	0.313
Pain (VAS)	5.2 ±1.6	5.1 ±1.6	0.888	0	0	1
TACOP (mm)	409.9 ± 111.3	440.9 ± 164.4	0.735	301 ± 26.7	314.4 ± 26.2	0.882
VCOP (mm/s)	14.1 ± 1.9	15.8 ± 3.3	0.071	10 ± 3	10.4 ± 2.6	0.735
LPC (mmHg)	19.1 ± 6.6	17.9 ± 10	0.722	10.5 ± 7.5	7.3 ± 5.2	0.304
Functionality (ODI)	31.3 ± 14.9	30.8 ± 11.7	0.950	0.55 ±0.4	2 ± 0.8	0.261

LBP, low back pain; SD, standard deviation; *, statistical significance; BMI, body mass index; IPAQ, International Physical Activity Questionnaire; VAS, visual analog scale; TACOP, total area of the center of pressure; VCOP, velocity of the center of pressure; LPC, lumbopelvic control; ODI, Oswestry Disability Index.

**Table 3:** Clinical variables by age across groups.

Variables	LBP Group			Control Group		
	20–40 y (*n* = 15) Mean ± SD	41–60 y (*n* = 15) Mean ± SD	*p*-Value (<0.05 *)	20–40 y (*n* = 15) Mean ±SD	41–60 y (*n* = 15) Mean ± SD	*p*-Value (<0.05 *)
BMI	24.52 ± 2.6	25.1 ± 3.6	0.619	23.9 ± 2.56	25.3 3 ± 3	0.278
IPAQ	1349.8 ± 323.5	1266.3 ± 360.3	0.432	4973 ± 2900	3460 ± 1822	0.180
Pain (VAS)	5.13 ± 1.50	5.13± 1.64	1	0	0	1
TACOP (mm)	430.9 ± 145.9	413.6 ± 123.7	0.729	279.9 ± 84.9	334 ± 73.7	0.143
VCOP (mm/s)	14.9 ± 3.2	14.6 ± 1.9	0.788	9.31 ± 2.8	11.1 ± 2.5	0.148
LPC (mmHg)	19.9 ± 9–03	17.2 ± 7.95	0.398	10.8 ± 7.7	7.2 ± 4.9	0.115
Functionality (ODI)	32..6 ± 14.19	29.47 ± 13.08	0.526	0.20 ± 0.6	2.2 ± 2.6	0.038 *

LBP, low back pain; SD, standard deviation; y, years; *, statistical significance; BMI, body mass index; IPAQ, International Physical Activity Questionnaire; VAS, visual analog scale; TACOP, total area of the center of pressure; VCOP, velocity of the center of pressure; LPC, lumbopelvic control; ODI, Oswestry Disability Index.

**Table 4:** Cohen’s d effect sizes and statistical power for variables comparing LBP subgroups and control group.

Variables	Male LBP vs. Control Group (*n* = 18) Cohen’s d (Power)	Female LBP vs. Control Group (*n* = 12) Cohen’s d (Power)	20–40 y LBP vs. Control Group (*n* = 15) Cohen’s d (Power)	41–60 y LBP vs. Control Group (*n* = 15) Cohen’s d (Power)
Pain (VAS)	4.60 (1.0)	4.51 (1.0)	4.84 (1.0)	4.42 (1.0)
TACOP (mm)	1.35 (0.99)	1.07 (0.98)	1.27 (0.99)	0.78 (0.84)
VCOP (mm/s)	1.63 (1.0)	1.82 (1.0)	1.86 (1.0)	1.58 (1.0)
LPC (mmHg)	1.22 (0.99)	1.33 (0.99)	1.08 0.98)	1.51 (0.99)
Functionality (ODI)	2.92 (1.0)	3.47 (1.0)	3.23 (1.0)	2.89 (1.0)

**Table 5:** Clinical variables by level of disability.

Variables	Mild (*n* = 7)	Moderate (*n* = 14)	Severe (*n* = 9)	*p*-Value (<0.05 *)
BMI	24.27 ± 0.9	25.2 ± 0.9	24.8 ± 1	0.8
IPAQ	1634.22 ± 623.3	1217.21 ± 259.5	1070.43 ± 425.5	0.66
Pain (VAS)	4.1 ± 1.1	5.14 ± 1.2	6.57 ± 2.2	0.002 *
TACOP (mm)	344.55 ± 74.8	419.75 ± 118.1	527.50 ± 161.3	0.018 *
VCOP (mm)	13.26 ± 2.2	15.19 ± 2.9	15.87 ± 1.7	0.105
LPC (mmHg)	13.88 ± 7.1	19.92 ± 2.6	21.85 ± 5.4	0.123
Functionality (ODI)	16.0 ± 2.8	31.43 ± 6.7	49.71 ± 5.1	0.001 *

*, statistical significance; VAS, visual analog scale; TACOP, total area of the center of pressure; VCOP, velocity of the center of pressure; LPC, lumbopelvic control; ODI, Oswestry Disability Index.

## Data Availability

The raw data supporting the conclusions of this article will be made available by the authors, without undue reservation.
